# Ganglioneuroma presenting as an adrenal incidentaloma: a case report

**DOI:** 10.1186/1752-1947-8-131

**Published:** 2014-04-29

**Authors:** Mine Adas, Bora Koc, Gokhan Adas, Filiz Ozulker, Tamer Aydin

**Affiliations:** 1 Department of Endocrinology, Okmeydani Training and Research Hospital, 34200 Şişli, İstanbul, Turkey; 2 Department of Surgery, Okmeydani Training and Research Hospital, 34200 Şişli, İstanbul, Turkey; 3 Department of Surgery, Bakırkoy Training and Research Hospital, 34700 Bakırkoy, İstanbul, Turkey; 4 Department of Nuclear Medicine, Okmeydani Training and Research Hospital, 34200 Şişli, İstanbul, Turkey; 5 Department of Pathology, Okmeydani Training and Research Hospital, 34200 Şişli, İstanbul, Turkey

**Keywords:** Adrenal ganglioneuroma, Adrenal gland, Incidentaloma

## Abstract

**Introduction:**

Ganglioneuromas are rare benign tumors arising from the neural crest tissue and are most commonly located in the posterior mediastinum and retroperitoneum; they are rarely found in the adrenal gland. This tumor is usually asymptomatic and in the majority of cases is detected incidentally. Although the characteristics of adrenal ganglioneuroma on computerized tomography and magnetic resonance imaging have been well described, the exact diagnosis is difficult. Histopathological examination is currently the mainstay of diagnosis. Ganglioneuromas have a very good prognosis with surgical removal. We report the case of a male patient with an incidentally identified adrenal ganglioneuroma with high standardized uptake values in a positron emission tomography scan.

**Case presentation:**

An 18-year-old Turkish male patient with no previous comorbidities was admitted to our hospital with lower-quadrant pain. He had no significant past medical or surgical history. A physical examination did not reveal any signs and the results of routine laboratory tests were all within the normal ranges. Our patient underwent computed tomography of his abdomen, which showed a relatively homogenous left adrenal tumor measuring 5.2×4.3×7.1cm. On a positron emission tomography scan, the left adrenal gland disclosed a standardized uptake value of 4.1. Our patient underwent an exploratory laparotomy with left adrenalectomy without any related complications.

**Conclusion:**

Ganglioneuroma may sometimes be similar to other adrenal malignancies. Careful evaluation with endocrine tests and imaging procedures is necessary to provide an accurate diagnosis. Definitive diagnosis can be made by histological examination. The prognosis is very good with surgical removal.

## Introduction

Ganglioneuroma (GN) is a rare benign tumor arising from the neural crest tissue and most commonly located in the posterior mediastinum and retroperitoneum. GN’s are rarely found in the adrenal gland [1,2]. This tumor is usually asymptomatic and, in the majority of cases, detected incidentally. Now that imaging procedures such as ultrasonography and computerized tomography (CT) are used more frequently, this unexpected and exceptional incidentaloma lesion can be detected more easily [3,4]. The prevalence of adrenal incidentaloma is 0.2% in young patients, 3% in populations past their fifth decade, and as high as 7% in those past their seventh decade [5].

Although adrenal GN’s are usually hormonally inactive, some of them secrete catecholamines. Symptoms like hypertension, diarrhea and virilization may develop as a result of mixed hormone secretion; in such case the diagnosis will be controversial. Tumors that originate from the ganglion cells include GN (benign), ganglioneuroblastoma (intermediate differentiation) and neuroblastoma (highly malignant lesion). The imaging characteristics of adrenal GN are variable and some are very similar to other adrenal tumors such as adrenocortical carcinoma (ACC) and pheochromocytoma, a fact that is crucial in the clinic [4,6]. Therefore, it is generally challenging to obtain a precise differential diagnosis of adrenal GN prior to surgery. A definitive diagnosis can be made by histological examination.

In this case report, we present the case of an 18-year-old male patient with an adrenal tumor. The tumor was found incidentally and was diagnosed as an adrenal GN during an evaluation of his right lower-quadrant pain.

## Case presentation

An 18-year-old Turkish male patient with no previous comorbidities was admitted to our hospital with right lower-quadrant pain. He had no significant past medical or surgical history. A physical examination revealed no signs and the results of routine laboratory tests were all found to be within the normal ranges. Because of his symptoms, an abdominal ultrasound was performed and showed a heterogeneous, well-defined mass measuring 5×7cm at his left adrenal gland. Our patient was referred to the endocrinology department where he underwent abdominal CT, which showing a relatively homogenous left adrenal tumor measuring 5.2×4.3×7.1cm with faint calcification and well-defined edges. Magnetic resonance imaging (MRI) showed a solid mass measuring 5×4×7cm arising from his left adrenal gland. The tumor was slightly hypointense on T1A-weighted MRI, whereas it was slightly hyperintense on T2A-weighted MRI. After an intravenous injection of gadolinium, the mass showed a progressive, heterogeneous and delayed enhancement.

**Figure 1:**
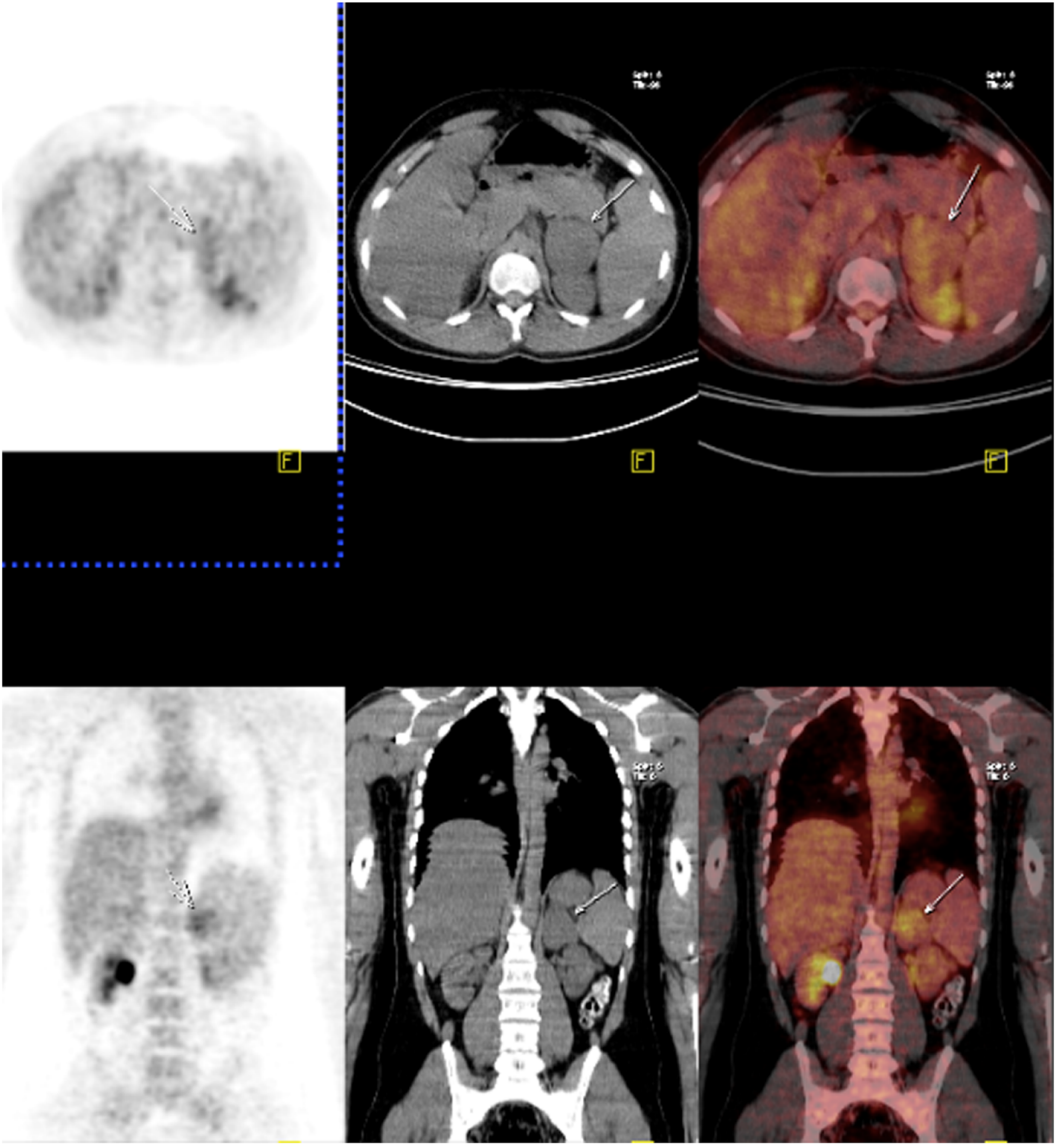
**Positron emission tomography and computerized tomography scans of the patient with a left adrenal tumor. **^18^F-2-fluoro-deoxy-D-glucose-positron emission tomography scans show a standard uptake value of 4.1 for a left adrenal mass measuring 5.2×4.3×7.1cm. (arrow shows the mass on computed tomography).

An endocrine workup, including urine catecholamine and cortisol levels and a 1mg overnight dexamethasone suppression test, was normal. Because of the tumor size, we performed an ^18^F-2-fluoro-deoxy-D-glucose-positron emission tomography (PET) scan to diagnose the malignant lesion. His left adrenal gland showed a standardized uptake value (SUV) of 4.1 (Figure 1). With these findings, it was not possible to conclude whether the adrenal tumor was benign or malignant.

**Figure 2:**
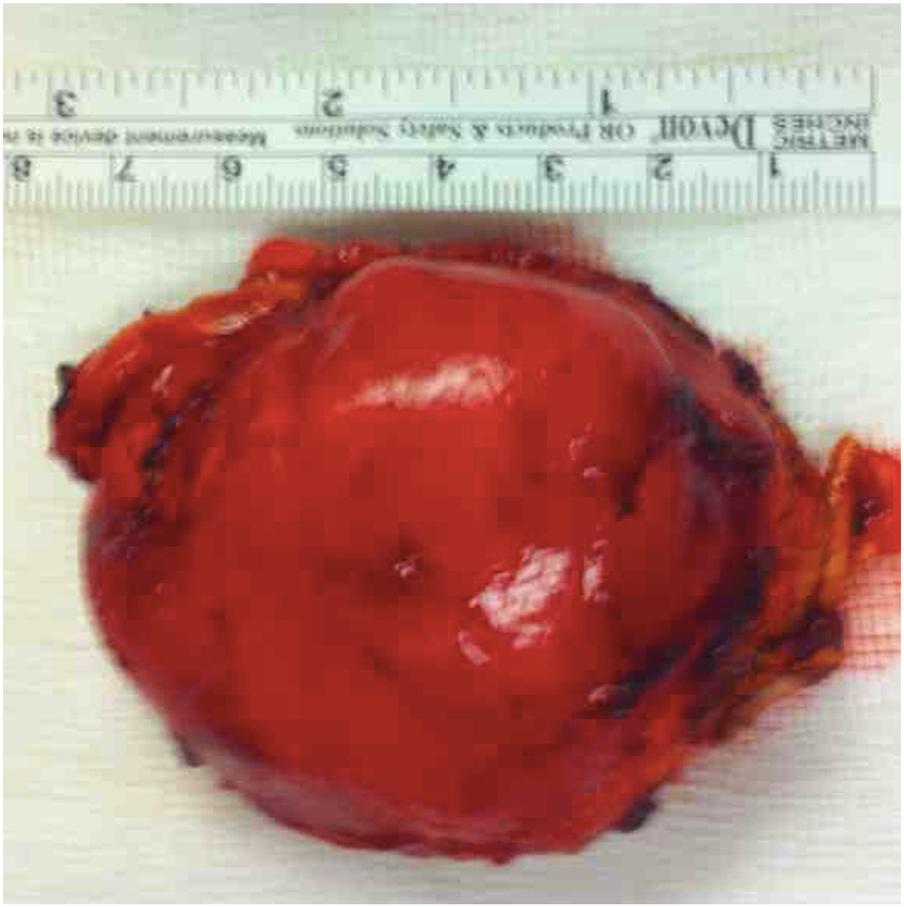
**Macroscopic view of the specimen.** Surgical specimen was an elastic tumor with a slightly lobular edge, measuring 4.4×5.1×7.3cm.

**Figure 3:**
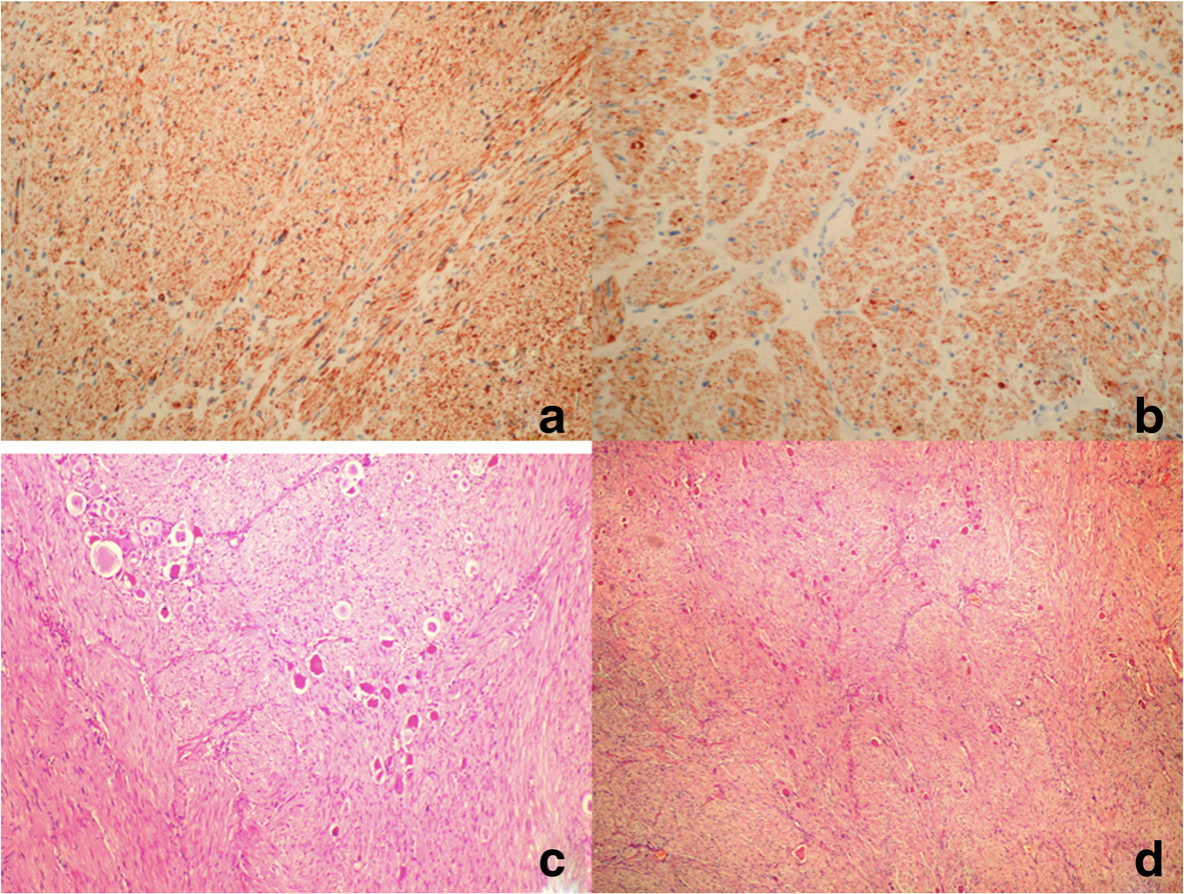
**Immunohistochemical examination of the mass. (a)** Positive staining for vimentin, ×200. **(b)** Positive staining for synaptophysin, ×200. **(c)** Pattern of nerve bundles in the neoplasm with vesicular nucleus and huge ganglion cells including wide cytoplasm. Hematoxylin and eosin staining, ×40. **(d)** Ganglion cell group in detail. Hematoxylin and eosin staining, ×100.

For this reason, exploratory laparotomy was performed to allow a definite diagnosis. A left adrenalectomy was performed, with no related complication. The surgical specimen was an elastic tumor with a slightly lobular edge, measuring 4.4×5.1×7.3cm (Figure 2). The cut surface of the tumor was light brown, covered by a thin capsule without any evidence of hemorrhage or necrosis. On microscopy, the section showed irregular proliferation of spindle-shaped cells and scattered mature ganglionic cells with dystrophic changes and focal lymphocytic infiltration. No evidence was found for the malignancy. An immunohistochemical examination showed positive staining of the ganglion and Schwann cells for S-100, vimentin and synaptophysin (Figure 3). The tumor was diagnosed as a left adrenal GN. Our patient experienced no complications during his postoperative course. No recurrence was detected during the one-year follow-up visits.

## Discussion

GN is a rare, differentiated and benign tumor arising from primordial neural crest cells that form the sympathetic nervous system [7]. On histology, it is composed of mature Schwann cells and ganglion cells with fibrous stroma [1,8]. GN is a member of a group of neurogenic tumors group that includes ganglioblastoma and neuroblastoma. It differs from other neurogenic tumors in its benign potential [7]. These non-functional and non-symptomatic masses are usually detected incidentally and are referred to as incidentaloma. Because of the improvements in CT and ultrasonography procedures, the number of incidentalomas found has recently increased.

The pathology of adrenal incidentalomas may vary from a simple benign cyst or lipoma to adrenal carcinoma. The differential diagnosis of an adrenal mass comprises a long list including adenoma, myelolipoma, cyst, lipoma, pheochromocytoma, adrenal cancer, metastatic cancer, hyperplasia and tuberculosis [1-8]. Functioning tumors and carcinomas are not generally discovered incidentally because their diagnosis is based on specific signs and symptoms. It should be noted that some functional tumors may present a subclinical form without any specific symptoms and some malignant tumors may look like benign lesions in radiological evaluations. The diagnosis of an adrenal tumor as a pheochromocytoma depends on the ability to determine its catecholamine content by biochemical or histochemical methods. Although extra-adrenal tumors have also been designated as pheochromocytomas by some authors, the preferred terminology for such neoplasms is ‘extra-adrenal paraganglioma’. Terms such as ‘composite pheochromocytomas’ and ‘compound tumor of the adrenal medulla’ have been used to describe tumors containing pheochromocytoma together with foci of neuroblastoma, ganglioneuroblastoma, GN or malignant peripheral nerve sheath tumor [9]. Literature shows that about 71% of composite pheochromocytoma or paraganglioma coexist with GN. Like ordinary pheochromocytomas and paragangliomas, most cases of composite pheochromocytomas or paragangliomas were functional, with increased levels of catecholamines or corticotropin-releasing hormone [10].

Although GN is generally considered to occur more frequently in young people, some recent studies have shown that it may also be seen between the ages 40 and 50 [4,11]. GN is most commonly found in the posterior mediastinum and retroperitoneum, and the involvement of the adrenal gland is relatively rare (21%) [12]. Adrenal GN is usually regarded as having silent hormonal functions and therefore can be asymptomatic. Occasionally, composite tumors with pheochromocytoma are seen and they, rarely, can secrete cortisol and androgen [13,14]. Additionally, Geoerger *et al*. reported that up to 30% of patients with GN had elevated plasma and urinary catecholamine levels but that patients were rarely found to have symptoms of catecholamine excess [1]. By contrast, Koch *et al*. reported the case of a patient with a GN that was positive for vasoactive intestinal peptide, which is the product of ganglion cells [15]. Patients with vasoactive intestinal peptide-positive tumors such as GN and neuroblastomas may not have any symptoms or signs of vasoactive intestinal peptide secretion [16]. GN can also produce and secrete other hormones, such as testosterone, indicating the pluripotency of its precursor cell. In our patient, his tumor was hormonally inactive and asymptomatic [17].

GN is a benign neoplasm that originates from neural crest cells of sympathetic ganglia or adrenal medulla. The macroscopic characteristics of adrenal GN are an encapsulated mass with a firm consistency and a solid, homogeneous, grayish-white cut surface. Histopathological examination shows mature ganglion cells and Schwann cells among a fibrous stroma. Using microscopy they can be classified in two main groups: mature and maturing. The mature type is composed of mature Schwann cells, ganglion cells and perineurial cells, whereas the maturing type consists of cells with different maturation levels, ranging from mature cells to neuroblasts with a similar stroma. According to immunohistochemical analysis, GN are characterized by reactivity for S-100, vimentin, synaptophysin and neuronal markers [14]. In our patient, a final pathological examination confirmed the diagnosis.

Although the imaging characteristic of adrenal GN on CT and MRI have been well described, the precise diagnosis of adrenal GN using radiological evaluation prior to surgery is difficult. Qing *et al*. reported that the misdiagnosis rate of adrenal GN on CT and MRI before surgery is 64.7% [4]. Adrenal GN is described as a well-circumscribed tumor with lobular shape and low attenuation. In the literature, intratumoral calcification has been determined in 0% to 29% of the cases [4,6]. On MRI, adrenal GN shows homogenously low or intermediate signal intensity in T1-weighted images and heterogeneous slightly high signal intensity on T2-weighted images [14]. For a tumor size larger than 5cm, heterogeneity and calcification may suggest malignancy [18]. In our patient, because the size and heterogeneity of the mass made us suspect malignancy, we preferred to perform a PET scan prior to the surgery.

PET scans can help complete the picture obtained by CT and MRI when making a differential diagnosis between adrenal GN and ACC or metastasis. One study reported that all cases of ACC had a SUV of 3.0 or higher, and that the sensitivity and specificity to distinguish ACC from adenoma were 100% and 98%, respectively [19,20]. In another review of four patients with adrenal GN, the SUVs were between 1.5 and 2.9 [11]. The SUV in our case was 4.1, which also suggested ACC. A PET scan is one of the most helpful modalities to differentiate malignancy and adenoma. However, in rare cases it may still mislead the physicians.

Even though a lesion size greater than 4.5cm seems to be a strong predictor of malignancy, this is not correctly confirmed by histologic evaluations. In our patient, although his tumor was larger than 6cm and the SUV level on PET was above than 3.0, pathology confirmed a benign tumor. Recent studies recommended that non-secretary adrenal incidentalomas larger than 6cm or with suspicious features of malignancy on imagining procedures should be treated by adrenalectomy [3-7]. There is no medical treatment for such tumors. GN, although benign, can grow aggressively. Patients treated surgically for a benign neurogenic tumor have an excellent prognosis. Papavramidis *et al*. reported that adrenal GN should be resected by adrenalectomy, whereas retroperitoneal GN can be resected without adrenalectomy [21]. The role of laparoscopic adrenalectomy in patients with large adrenal lesions or potential malignancy remains controversial. The reduced hospital stay and morbidity have resulted in laparoscopic adrenalectomy becoming the procedure of choice for the surgical removal of a vast majority of small sized (<6cm) adrenal lesions [1,3,7]. The prognosis for an adrenal GN following surgical resection is good without the need for additional treatment. A few cases of recurrence have been reported. Long-term follow-up is recommended.

## Conclusion

An adrenal GN is a rare, hormonally silent benign tumor. GN can sometimes resemble other adrenal malignancies. Careful evaluation by endocrine tests and imaging procedures is necessary for an accurate diagnosis. A definitive diagnosis can be made by histological examination. The prognosis is very good with surgical removal.

## Consent

Written informed consent was obtained from the patient for publication of this care report and any accompanying images. A copy of the written consent is available for review by the Editor-in-Chief of this journal.

## Abbreviations

ACC: adrenal cortical carcinoma; CT: computer tomography; GN: ganglioneuroma; MRI: magnetic resonance imaging; PET: positron emission tomography; SUV: standard uptake value.

## Competing interests

The authors declare that they have no competing interests.

## Authors’ contributions

MA and BK took care of patient and wrote the initial draft. BK and GA operated on the patient. TA performed the pathologic evaluation of the specimen. BK, GA and FO edited the manuscript and performed the literature review. All authors read and approved the final manuscript.
